# Allelopathic Effects of *Lantana camara* L. Leaf Aqueous Extracts on Germination and Seedling Growth of *Capsicum annuum* L. and *Daucus carota* L.

**DOI:** 10.1155/2024/9557081

**Published:** 2024-04-16

**Authors:** Yiftusira Alemayehu, Meseret Chimdesa, Zekeria Yusuf

**Affiliations:** School of Biological Sciences and Biotechnology, Haramaya University, Dire Dawa, Ethiopia

## Abstract

Allelopathy is the chemical interactions between plants that might lead to either stimulation or inhibition of growth, community structure, and plant invasions. *Lantana camara* L. is a noxious invasive weed that negatively affects seed germination, seedling growth, and increases the mortality of the crop plant. The objective of this work was to assess allelopathic effect of *L. camara* leaf aqueous extract on germination and seedling growth of *Capsicum annuum* (pepper) and *Daucus carota* (carrot). The aqueous extract of Lantana leaf samples was used as a source of allelopathic effects. Data were collected for germination and seedling growth parameters. The result indicated that the highest concentration of the allelopathic extract (20 mg/L) has demonstrated significantly the highest germination inhibition rate GIR (60.00%), germination speed V (2.54 U/day) for *D. carota* as GIR (70.00%), mean germination time MGT (0.36 days), and GI (0.67%) for *C. annuum* seeds. The highest concentration of the allelopathic extract (20 mg/L) has recorded the highest plumule inhibition rate PIR (59.63%) and radical inhibition rate RIR (48.95%) for *D. carota* seeds, as well as PIR (27.47%) and RLR (79.49%) for *C. annuum*. The largest negative allelopathic index (−60.00% or allelopathic intensity of 60.00%) was recorded for *D. carota* seeds, whilst (−63.43% or allelopathic intensity of 63.43%) was recorded for *C. annuum* seed germination. For *D. carota* seed germination, the first principal component (PC1) has got high positive loads from GI (0.36), RLR (0.31), GR (0.34), allelopathic index AI (0.34), relative length of plumule RLP (0.24), and V (0.30). By contrast, PC1 for *D. carota* seed germination has got the highest negative component loads recorded by GIR (−0.34), PIR (−0.24), MGT (−0.35), and RIR (−0.31). In allelopathic effect on *C. annum* seed germination, the first principal component (PC1) has got high positive scores from relative length of radical RLR (0.31), RLP (0.33), germination rate GR (0.33), V (0.33), and AI (0.33). Likewise, the high negative component loads were recorded by GIR (−0.33), PIR (−0.33), RIR (−0.31), and MGT (−0.32). The result of the present study demonstrated that GIR, PIR, and RIR were directly related to negative allelopathic activity.

## 1. Introduction

The term *allelopathy* derived from Greek words, namely, *allele* and *pathy* (meaning “mutual harm” or “suffering,” respectively) [1]. Allelopathy is the interaction between plants in neighbourhood that might lead to either stimulation or inhibition of growth by allelochemicals that are released through volatilization, eluviations, and decomposition of the plant or root exudates during growth [2]. Allelochemicals can also indirectly affect plants through the inhibition of microorganisms, including nitrogen fixing and nitrifying bacteria [3] and ectomycorrhizae [4]. Allelopathy widely exists in nature and plays a vital role in crop cultivation systems, controlling weeds, and preventing crop disease and insect infection. Different groups of plants, like algae, lichens, crops, and annual and perennial weeds have widely known allelopathic interactions [4, 5].

A significant portion of the agricultural land in developing countries particularly tropics is heavily infested by various native and alien (invasive) weeds [6], and controlling weeds is a big challenge to farmers [7]. The crop-weed interaction can be owing to competition alone, allelopathy, or both [8]. Many allelochemicals are phytotoxic and have potential as herbicides or as templates for new herbicide classes. Allelopathic effects can be useful in some environmentally friendly techniques for controlling the weeds and help reduce the economic and environmental costs of using herbicides because herbicides create environmental pollution [9, 10]. Various reports of allelopathic interactions also reported including bioactive compounds [11], responses of plant proteomes to soil antibiotics [12], biofungicide [13, 14], and biopesticide [15].

Allelochemicals can stimulate or inhibit the germination or/and growth of plants and increase the resistance of crops to biotic and abiotic stresses [2]. Allelochemicals are mainly secondary metabolites present in different parts of plants. Chemicals that inhibit the growth of some species at certain concentrations can stimulate the growth of the same or different species at lower concentrations [16]. The allelopathic nature of the plants help them to be highly competitor for space, light, and nutrients with the nearby plants [9]. The allelochemicals are released into the neighboring environments and rhizosphere soil of the plants and neighbouring environments during rainfall leachates, decomposition of plant residues, root exudation, and volatilization from living plant parts [17–19]. The released allelochemicals may suppress the regeneration process of indigenous plant species by decreasing their germination and seedling growth and increasing their mortality. The natural decomposition process of crop residues induced by microbes, dispels chemicals in soil which are potentially very toxic even though the primary substances are not toxic [20].

Allelopathic plants inhibit or suppress germination, growth, development, or metabolism of crops due to secretion of allelochemicals into the rhizosphere of neighboring crop plants [21]. Various phenolic compounds inhibited cell division. It is also possible that cell elongation was affected by extracts of weed residues. Many phytotoxic allelochemicals have been isolated, identified, and found to influence a number of physiological reactions. These allelochemicals affected many cellular processes in target plant species, including disruption of membrane permeability [22], ion uptake [23], inhibition of electron transport in both photosynthesis and the respiratory chain [24], damage to DNA and protein, alterations of some enzymatic activities [25, 26], and ultimately leading to programmed cell death [25].

*Lantana camara* is an invasive weed belonging to Verbenaceae family [27]. It interrupts the regeneration process of indigenous plant species by decreasing their germination, reducing their seedling growth, and increasing their mortality [27, 28]. The extracts, essential oil, leachates, residues, and rhizosphere soil of *L. camara* suppressed the germination and growth of other plant species [28, 29]. Therefore, the allelopathic property of *L. camara* may support its invasive potential and the formation of dense monospecies stands [29].

The elevated temperature has been contributing to the allelopathy of *L. camara* [30], which indicates that global warming may increase the threat of the invasion of the species into additional nonnative areas. Direct negative allelopathic effects of *Lantana* spp. on some crops have been reported previously [5, 8, 31]. However, these effects may vary depending on the species variety. Farmers in the Hararghe region of eastern Ethiopia cultivate different vegetables including carrot and pepper for which information regarding the allelopathic effect of *Lantana* spp. is insufficient. Therefore, this research was initiated to evaluate the allelopathic effect of Lantana leaf extract on germination and seedling growth of *Capsicum annuum* and *Daucus carota.*

## 2. Materials and Methods

### 2.1. Sample Collection and Preparation

The experiment was conducted at Botany laboratory of the School of Biological Sciences and Biotechnology, Haramaya University. The lantana leaf sample was collected from farmer's field in Bate locality, Haramaya district, Oromia regional state, Ethiopia. The *Capsicum annuum* and *Daucus carota* seed samples were obtained from Raare Research Station, Haramaya University.

### 2.2. Preparation of the Aqueous Extracts

The leaf sample was air dried at room temperature for 10 days and dried leaf sample was ground to fine powder by using mortar and pestle. Then aqueous extraction was done with distilled water by dissolving 120 grams of the powder in 500 mL of distilled water in a 500 ml flask. The extract was then filtrated through Whatman no. 1 filterpaper, and the filtrate was dried in rotary evaporator at 50°C under reduced pressure to evaporate water and obtain the extracts in a somewhat dried form [32]. Aqueous extracts of different concentrations (10, 15, and 20 mg/L) were then prepared by dissolving the dried crude extract in distilled water. The control experiment, without the use of the aqueous extract was made with distilled water. The crude extract was stored at 4°C until use. All experiments were replicated three times.

### 2.3. Seed Germination and Seedling Growth Bioassays

The experiment was carried out in sterile 20 cm diameter Petri dishes, in which moistened Whatman No. 3 paper was used as a germination support. Before being used, the tested seeds were chosen as healthy and then disinfected with 70% ethanol. Afterwards, 10 seeds were placed in each Petri dish before being treated with a sufficient amount of various concentrations of the aqueous extract, while the control was only treated with distilled water. The experiment was carried out under laboratory conditions for a period of 2 months in three replications for each treatment. A seed is considered germinated when the radicle appears. Germination was recorded daily, and the results were determined by measuring different germination and seedling growth parameters like root length (cm), number of leaves, and seedling dry weight. Germination parameters like germination kinetics, germination index, inhibition rate, and the allelopathic index (AI) were calculated as follows:

#### 2.3.1. Germination Rate (GR)

The germination rate was calculated according to the formula given by Come [33]:

(1) $$\begin{array}{c} \text{GR}c\left(\%\right)=\frac{Ng}{Ns}\;x\;100, \end{array}$$

where *Ng* is the number of germinated seeds and *Ns* is the number of seeds sown.

#### 2.3.2. Germination Speed (V)

The germination speed is calculated by the following formula proposed by Come [33]:

(2) $$\begin{array}{c} V=\frac{N1+N2+N3\;+,\cdots ,+\;Nn}{N1T1+N2T2+N3T3\;+,\cdots NnTn}, \end{array}$$

where *V* is the speed of germination and *N*1 is the number of seeds germinated at time *T*1.

#### 2.3.3. Mean Germination Time (MGT)

The mean germination time is calculated as described by Ranal et al. [34]:

(3) $$\begin{array}{c} \text{MGT}=\frac{\sum_{i=1}^{k}niti}{\sum_{i=1}^{k}ni}, \end{array}$$

where *n*_*i*_ and *t*_*i*_ are consecutively the numbers of newly germinated seeds in the last time and the time from the beginning of the experiment to the last observation and *k* is the last time of germination.

#### 2.3.4. Germination Index

The germination index (GI) is a quantitative expression of germination which relates to the daily germination rate at the maximum value of germination [35], it is calculated by the following equation:

(4) $$\begin{array}{c} N1+GI=\frac{N2-N1}{2}+\frac{N3-N2}{3}+\frac{N_{n}-N_{n-1}}{n}, \end{array}$$

where *N*_*n*_ is the percentage of germination on *n*^th^ day.

#### 2.3.5. Relative Length of Radicles (RLR)

The relative length of radicles is calculated according to the formula given by Rho and Kil [36]:

(5) $$\begin{array}{c} \text{RLR}=\frac{Lr}{Lc}\;x\;100, \end{array}$$

where Rr is the relative length of radicle; Lr is the average length of radicles of treated plants and Lc is the average length of radicle of control plants.

#### 2.3.6. Relative Length of Plumule (RLP)

According to Rho and Kil [36] this parameter is calculated by the following formula:

(6) $$\begin{array}{c} \text{RLP}=\frac{Lp}{Lc}\;x\;100, \end{array}$$

where Rs is the relative length of plumule; Lp is the average length of plumule of treated plants; and Lc is the average length of plumule of control plant.

#### 2.3.7. The Growth Inhibition Rate

This parameter is calculated according to the following formula given by Abiyu et al. [37]:

(7) $$\begin{array}{c} \text{PIR}\left(\%\right)=100-\text{RLP},\text{for inhibition of plumule length},\\ \text{RIR}\left(\%\right)=100-\text{RLR},\text{for inhibition of radicle length}. \end{array}$$

where PIR: plumule inhibition rate; RIR: radicle inhibition rate.

#### 2.3.8. Allelopathic Index (AI) to Reflect the Intensity of the Allelopathic Effect

AI was calculated according to the work of Luo et al. [38] as follows:

(8) $$\begin{array}{c} \text{AI }\left(\%\right)=\left\lceil \frac{\left(\text{GR}t-\text{GR}c\right)}{\text{GR}c}\right\rceil \;x\;100, \end{array}$$

where GR*t* is the germination rate in treatment or allelopathic extract concentration *t* and GR*c* is the germination rate of the control (given sterile distilled water).

An AI < 0 reflects a negative allelopathic effect of *L. camara* leaf extract on seed germination, and an AI > 0 value indicates a promoting effect on the germination, while the absolute value of AI indicated the allelopathic intensity.

### 2.4. Statistical Analysis

All the experiments were performed in a completely randomized design. Data were subjected to one-way analysis of variance and were presented as mean separated at *p* < 0.05 applying least significant difference (LSD) test and statistical analysis was conducted using SAS version 9.2 software package.

## 3. Results

### 3.1. Allelopathic Effect of *Lantana camara* Leaf Aqueous Extract on Germination and Seedling Growth of *D. carota* and *C. annuum* Seeds

The impacts of *L. camara* leaf extracts on seed germination of *D. carota* and *C. annum* were assessed using germination parameters including germination rate (GR), germination inhibition rate (GIR), germination speed (V), mean germination time (MGT), and germination index (GI) (Table 1). For both bioassayed plants the control group has recorded significantly the highest GR (100% for *D. carota,* and 83.33% for *C. annum*); germination speed (2.40 U/day for *D carota,* and 8.33 U/day *C. annuum*); and GI (16.89% for *D. carota,* and and 0.61% for *C. annuum*). On the other hand, the maximum concentration of the extract has demonstrated significantly the highest GIR (60% for *D. carota*, and 70% for *C. annuum*); MGT (3.83 for *D. carota*, and 0.36 for *C. annuum*) indicating that GIR and MGT of the extracts increased with increasing extract concentration. Thus, MGT and GIR can be recommended as the best indicators of allelopathic effect of seed germination if corroborated with further works.

### 3.2. Effect of *L. camara* Aqueous Leaf Extract on Seedling Growth of *D. carota* and *C. annuum*

The allelopathic effect of *L. camara* leaf extract on seedling growth of both bioassayed plants including *D. carota* and *C. annuum* are shown in Table 2. The least RLP (40.37%) and RLR (51.05%) were recorded for *D. carota*; RLP (72.53%) and RLR (29.51%) for *C. annuum* were recorded with maximum concentration (20 mg/L) of *L. camara* leaf extract. The reduction in the relative growth of both plumule and radicle with increase in concentration of the extract might indicate the growth inhibitory effect of allelochemicals in the crude leaf extract of *L. camara*. The highest PIR (59.63%), RIR (48.95%), negative allelopathic index or allelopathic intensity (ǀAIǀ) (60.00) for *D. carota*; PIR (27.47%), RIR (70.49%), and allelopathic intensity (ǀAIǀ) (63.43) for *C. annuum* were recorded with the maximum concentration of the allelopathic extract (20 mg/L) indicating that PIR, RIR, and ǀAIǀ are the best parameters to measure the negative allelopathic effect on seed germination and seedling growth of crop plants.

### 3.3. Allelopathic Index for *D. carota* and *C. annum* Seed Germination

**Figure 1:**
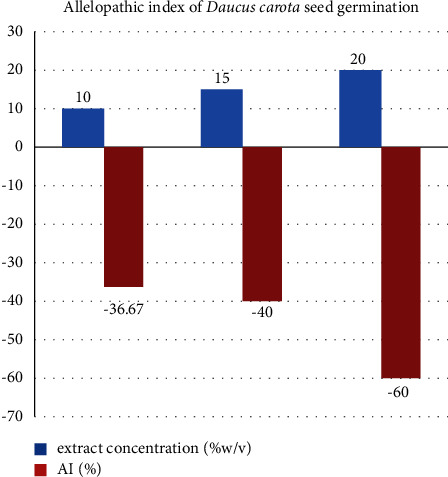
Allelopathic index (AI) of *D. carota* seed germination.

**Figure 2:**
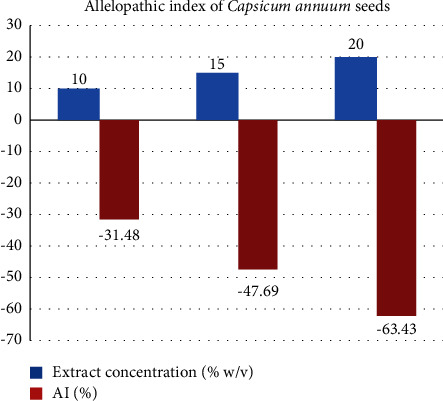
Allelopathic index (AI) of *Capsicum annuum* seeds.

The allelopathic index (AI) for *D. carota* seed germination (Table 2 and Figure 1) and *C. annuum* seed germination (Table 2 and Figure 2) demonstrated negative allelopathic activity as the concentration of the allelopathic extract from *L. camara* leaf increases from 10 to 20 mg/L. The allelopathic intensity was found to be the highest (60%) for *D. carota* and (63.43%) for *C. annuum* seed germination (Table 2). The large negative allelopathic activity of *L. camara* leaf extract might contribute to the retardation of growth and yield loss of crop plants near *L. camara* invasive shrub.

### 3.4. Correlation of Germination Parameters and Allelopathic Index of *L. camara* Leaf Extract

The Pearson's correlation coefficient was used to assess the association of *D. carota* and *C. annuum* seed germination parameters and the allelopathic index of *L. camara* leaf aqueous extract (Tables 3 and 4). The negative allelopathic index (AI) for *D. carota* seed germination (Table 3) was significant and negatively correlated with RIR (−0.762), GIR (−1.00), and germination speed (−0.807), indicating that the negative allelopathic activity increases with increasing RIR, GIR, and germination speed. However, the allelopathic index was found to be significant and positively correlated with RLR (0.762), germination rate (1.00), MGT (0.909) and germination index (0.944), indicating that the negative allelopathic activity decreases with increasing rate of germination, germination index, MGT, and RLR during *D. carota* seed germination. Thus, germination inhibition rate (GIR), radicle inhibition rate (PIR), and germination speed were found to be the best parameters or traits that can be used as indicators of allelopathic activity during *D. carota* seed germination.

The negative allelopathic index (AI) for *C. annuum* seed germination (Table 4) was significant and negatively correlated with plumule inhibition rate (−0.903), radicle inhibition rate (−0.835), germination inhibition rate (−0.987), and the mean germination time (−0.922), indicating that the negative allelopathic activity increases with increasing PIR, RIR, GIR, and MGT in *C. annuum* seed germination. Contrastingly, the allelopathic index was found to be significant and positively correlated with RLP (0.904), RLR (0.835), germination rate (0.987), and germination speed (0.987), indicating that the negative allelopathic activity decreases with increasing rate of relative length of plumule, relative length of radicle, germination rate, and germination speed.

Since the correlation coefficient indicated the existence of association among traits. It does not indicate the direct and indirect relationships among them. Therefore, the principal component analysis (PCA) was used to identify the most discriminating traits showing allelopathic activity of the plant extract (Table 5). The eigen values corresponding to sum of squares of variances greater than one being accounting for the majority of the variances were considered for interpretation of the result [39]. The PCA of allelopathic activity of *L. camara* on seed germination of *D. carota and C. annum* (Table 5) demonstrated that only PC1 (with eigenvalue >1) account for 95% of the variances for *D. carota*, while for *C. annum* seed germination, PC1 (with eigen value > 1) accounted for about (99%) of the variations; hence, the first PC was sufficient for interpretation of the relationship allelopathic index and germination parameters.

For *D. carota* seed germination, the first principal component (PC1) has got high positive loads from GI (0.36), RLR (0.31), GR (0.34), AI (0.34), RLP (0.24), and V (0.30). By contrast, PC1 for *D. carota* seed germination has got the highest negative component loads recorded by GIR (−0.34), PIR (−0.24), MGT (−0.35), and RIR (−0.31). The negative loads indicate that the negative allelopathic activity increases with increasing germination inhibition rate, plumule, and radicle inhibition rates. As the positive loads indicate that the positive allelopathic index increases with increment of germination rate, the relative length of plumule, the relative length of root, and the germination index in *D. carota* seed germination.

In allelopathic effect on *C. annum* seed germination, the first principal component (PC1) has got high positive scores from RLR (0.31), RLP (0.33), GR (0.33), and V (0.33) and AI (0.33). Likewise, the high negative component loads were recorded by GIR (−0.33), PIR (−0.33), RIR (−0.31), and MGT (−0.32). Therefore, the highest negative scores in PCs indicate direct relationship between the negative allelopathic index and negative score factors. However, the highest positive scores in PCs indicate inverse relationship between negative allelopathic activity and positive score factors in *C. annum* seed germination.

## 4. Discussion

The use of *Daucus carota* as a representative of storage root crops and *Capsicum annuum* as storage fruit vegetable so as to demonstrate differential response to the allelopathic effect of *L. camara.* It was found that for *D. carota* the inhibition on root is lower than that of shoot. However, for *C. annuum* inhibition of root elongation is higher than that of shoot. The fact that *D. carota* is a storage root crop might have made the growth of root more vigorous than shoot. However, it must be confirmed with further study. The maximum concentration of the allelopathic extract (20 mg/L) has demonstrated significantly the highest GIR and MGT indicating that GIR and MGT of the extracts increased with increasing extract concentration. This finding was in agreement with a number of previous studies like Maiti et al. [40] who reported the inhibitory effect of aqueous leaf extracts and leaf leachates of *L. camara* on seed germination metabolism of mungbean seeds. Similar result was also reported by Nandi and Dalal [41] the allelopathic effect of *L. camara* on germination and seedling growth of radish (*Raphanus sativus* L.) and spinach (*Spinacia oleracea* L.). Aqueous extracts of all parts of *Lantana camara* have strong allelopathic effect on the germination of *Pennisetum americanum*, *Lactuca sativa* and *Setaria italica* [42].

In the present study the allelopathic inhibitory effect was increasing with the concentrations of the extracts and higher concentration had the stronger inhibitory effect whereas the lower concentration showed stimulatory effect in some cases. The inhibitory effect was much in root than in shoot during seed germination. Similar finding was alsao reported by Ahmed et al. [4] who investigated the allelopathic effects of crop plants including *Brassica juncea* (L.) Czern; *Cucumis sativus* L.; *Phaseolus mungo* L.; *Raphanus sativus* L.; *Vigna unguiculata* (L.) Walp., and *Cicer arietinum* L. The allelochemicals released from *L. camara* may provide the species with a competitive advantage against the native plants and may also suppress the regeneration process of native plant species and contribute to establishing their habitats as invasive plant species and formation of monospecies stands [29].

According to Gniazowska and Bogtek [43] seed germination may also be inhibited due to hampered resource mobilization by allelochemicals during early stages of seed germination. It is also possible that allelochemicals such as some phenolic compounds impair the synthesis and/or activity of gibberellic acid [44], which regulates the production of amylase [45] so that seed germination is negatively affected. Based on the results of germination that tells us seed viability, *C. annuum* was more susceptible to the extracts than *D. carota.* This implies that different plant species may have varying sensitivity to allelochemicals of a given plant species.

The allelopathic effects of *L. camara* leaf extract on plumule and radicle growth is shown in Table 2. Compared to the lowest extract concentration, the lengths of plumule and radicle of the tested plants were significantly (*p* < 0.05) higher than the higher extract concentration treated seeds (Table 2). This finding agrees with that of Corsato et al. [46] and Rigon et al. [47] who, respectively, studied the allelopathic effects of leaf extracts of sunflower and castor on *Bidens pilosa* and, canola and raddish. Barreto et al. [48] also previously reported that radicle length of canola seedlings decreases with increasing concentration of soybean leaves extracts. They also explained that the length of the radicle is aindicator of the allelopathic effect as this plant part is very sensitive and in direct contact with the extracts. This observation of reduced initial seedling growth suggests that *L. camara* leaf extract possesses allelopathic compounds.

## 5. Conclusion

The present study has used *Daucus carota* as a representative of storage root crops and, *Capsicum annuum* as storage fruit vegetable so as to demonstrate differential response to the allelopathic effect of *L. camara.* It was found that for *D. carota* the inhibition on root is lower than that of shoot. However, for *C. annuum* inhibition of root elongation is higher than that of shoot. The fact that *D. carota* is a storage root crop might have made the growth of root more vigorous than shoot. However, it must be confirmed with further study. The allelopathic effect was found to be increasing as the concentration of the allelopathic extract increases from 10 to 20 mg/L for both *D. carota* and *C. annuum* seed germinations. The germination inhibition rate (GIR), radicle inhibition rate (PIR) and germination speed were found to be the best parameters or traits that can be used as indicators of allelopathic activity during *D. carota* seed germination. The the negative allelopathic activity increases with increasing PIR, RIR, GIR, and MGT in *C. annuum* seed germination. The highest negative scores in PCs indicate direct relationship between the negative allelopathic index and negative score factors while the highest positive scores in PCs indicate inverse relationship between negative allelopathic activity and positive score factors in *C. annum* seed germination. The large negative allelopathic activity of *L. camara* leaf extract might contribute for retardation of growth.

## Figures and Tables

**Table 1:** Allelopathic effect of *L. camara* leaf extract on germination of *D. carota* and *C. annuum* seeds.

Source	Allelopathic extract (mg/L)	GR (%)	GIR (%)	Germination speed (U/day)	MGT (days)	GI (%)
*D. carota*	0	100.00a	0.00c	2.40a	2.57c	16.89a
	10	63.33b	36.67b	1.29b	3.52b	1.30b
	15	60.00c	40.00b	0.80c	3.74a	1.02b
	20	40.00d	60.00a	0.54c	3.83a	0.48b

*C. annuum*	0	83.33a	16.67d	8.33a	0.12b	0.61a
	10	56.67b	43.33c	5.67b	0.18b	0.28b
	15	43.33c	56.67b	4.33c	0.23b	0.19c
	20	30.00d	70.00a	3.00d	0.36a	0.15c

GR, germination rate; GIR, germination inhibition rate; MGT, the mean germination time; GI, germination index.

**Table 2:** Allelopathic effect of *L. camara* leaf extract on seedling growth of *D. carota* and *C. annuum*.

	Allelopathic extract (mg/L)	RLP (%)	RLR (%)	PIR (%)	RIR (%)	AI (ǀAIǀ)
*D. carota*	10	85.79a	87.81a	14.21c	12.19c	−36.67a (36.67c)
	15	60.19b	61.71b	39.81b	38.29b	−40.00b (40.00b)
	20	40.37c	51.05c	59.63a	48.95a	−60.00c (60.00a)

*C. annuum*	10	91.02a	76.50a	8.98c	23.50c	−31.48a (31.48c)
	15	86.71ab	61.46b	13.30b	38.54b	−47.69b (47.69b)
	20	72.53b	29.51c	27.47a	70.49a	−63.43c (63.43a)

RLP, relative length of plumule; RLR, relative length of radicle; PIR, plumule inhibition rate; RIR, radicle inhibition rate; AI, allelopathic index; ǀAIǀ: negative allelopathic index or allelopathic intensity.

**Table 3:** Correlation coefficient of germination and allelopathic index of *D. carota*.

	RLS	RLR	PIR	PIR	GR	GIR	V	MGT	GI	AI
RLS	1.00	0.555	−1.00^*∗∗*^	−0.555	0.377	−0.377	−0.472	0.648	0.635	0.377
RLR		1.00	−0.555	−1.00^*∗∗*^	0.762^*∗∗*^	−0.762^*∗∗*^	−0.503	0.675^*∗*^	0.869^*∗∗*^	0.762^*∗∗*^
PIR			1.00	0.555	−0.377	0.377	0.472	−0.648	−0.635	−0.377
RIR				1.00	−0.762^*∗∗*^	0.762^*∗∗*^	0.503	−0.675^*∗*^	−0.869^*∗∗*^	−0.762^*∗∗*^
GR					1.00	−1.00^*∗∗*^	−0.807^*∗∗*^	0.909^*∗∗*^	0.944^*∗∗*^	1.00^*∗∗*^
GIR						1.00	0.807^*∗∗*^	−0.909^*∗∗*^	−0.944^*∗∗*^	−1.00^*∗∗*^
V							1.00	−0.899^*∗∗*^	−0.777^*∗∗*^	−0.807^*∗∗*^
MGT								1.00	0.934^*∗∗*^	0.909∗∗
GI									1.00	0.944^*∗∗*^
AI										1.00

GR, the germination rate; GIR, the germination inhibition rate; MGT, the mean germination time; GI, the germination index; RLP, the relative length of plumule; RLR, the relative length of radicle; PIR, the plumule inhibition rate; RIR, the radicle inhibition rate; AI, the allelopathic index. ^*∗*^Significant at *P* < 0.05; ^*∗∗*^significant at *P* < 0.01.

**Table 4:** Correlation coefficient of germination and allelopathic index of *C. annuum*.

	RLS	RLR	IRs	Irr	GR	GIR	V	MGT	GI	AI
RLP	1.00	0.883^*∗∗*^	−1.00^*∗∗*^	−0.883^*∗∗*^	0.872^*∗∗*^	−0.872^*∗∗*^	0.872^*∗∗*^	−0.882^*∗∗*^	−0.612	0.904^*∗∗*^
RLR		1.00	−0.883^*∗∗*^	−1.00^*∗∗*^	0.781^*∗∗*^	−0.781^*∗∗*^	0.781^*∗∗*^	−0.680^*∗*^	−0.57	0.835^*∗∗*^
PIR			1.00	0.883^*∗∗*^	−0.872^*∗∗*^	0.872^*∗∗*^	−0.872^*∗∗*^	0.882^*∗∗*^	0.612	−0.903^*∗∗*^
RIR				1.00	−0.781^*∗∗*^	0.781^*∗∗*^	−0.781^*∗∗*^	0.680^*∗*^	0.57	−0.835^*∗∗*^
GR					1.00	−1.00^*∗∗*^	1.00^*∗∗*^	−0.937^*∗∗*^	−0.595	0.987^*∗∗*^
GIR						1.00	−1.00^*∗∗*^	0.937^*∗∗*^	0.595	−0.987^*∗∗*^
V							1.00	−0.937^*∗∗*^	−0.595	0.987^*∗∗*^
MGT								1.00	0.614	−0.922^*∗∗*^
GI									1.00	−0.529
AI										1.00

GR, the germination rate; GIR, the germination inhibition rate; MGT, the mean germination time; GI, the germination index; RLP, the relative length of plumule; RLR, the relative length of radicle; PIR, the plumule inhibition rate; RIR, the radicle inhibition rate; AI, the allelopathic index. ^*∗*^Significant at *P* < 0.05; ^*∗∗*^significant at *P* < 0.01.

**Table 5:** The principal component analysis (PCA) for the allelopathic activity of *L. camara* leaf aqueous extract on germination of *D. carota* and *C. annum* seeds.

Parameters	PC1 (*D. carota*)	PC1 (*C. annuum*)
Eigenvalue	5.99	7.98
Difference	5.75	7.93
Proportion	0.95	0.99
Cumulative	0.95	0.99
RLP	0.24	0.33
RLR	0.31	0.31
PIR	−0.24	−0.33
RIR	−0.31	−0.31
GR	0.34	0.33
GIR	−0.34	−0.33
V	0.30	0.33
MGT	−0.35	−0.32
GI	0.36	−0.23
AI	0.34	0.33

GR, germination rate; GIR, germination inhibition rate; MGT, the mean germination time; GI, the germination index; RLP, the relative length of plumule; RLR, the relative length of radicle; PIR, the plumule inhibition rate; RIR, the radicle inhibition rate; AI, the allelopathic index.

## Data Availability

The data are included within the article and in the supplementary files.

## Additional Points

*Human and Animal Rights*. No humans and animals were used in this study. *Research Invloving Plants*. The plant species used in this study are not en-dangered.

## Supplementary Materials

Supplementary Materials Table S1: Raw data for allelopathic effect of *L. camara* leaf extract on germination of D. carota and C. annuum seeds; Table S2: Data for allelopathic effect of *L. camara* leaf extract on seedling growth of *D. carota* and *C. annuum* (supplementary information file).
