# Supplementary material for: Allelopathic Effects of Lantana camara L. Leaf Aqueous Extracts on Germination and Seedling Growth of Capsicum annuum L. and Daucus carota L
Source: Scientifica (Cairo). 2024 Apr 16;2024:9557081. doi: 10.1155/2024/9557081 (PMC11221968; doi:10.1155/2024/9557081)
Supplement: Supplementary Materials — Table S1: Raw data for allelopathic effect of L. camara leaf extract on germination of D. carota and C. annuum seeds; Table S2: Data for allelopathic effect of L. camara leaf extract on seedling growth of D. carota and C. annuum (supplementary information file). [file 9557081.f1.pdf]

Table S1. Allelopathic activity on germination of seeds

	19/06/15	20/06/15	21/06/15	22/06/15	23/06/15	24/06/15	25/06/15	26/06/15	27/06/15	28/06/15	29/06/15	30/06/15	30/06/15	1/6/2015	2/6/2015	3/6/2015	4/6/2015	5/6/2015	4/6/2015	4/6/2015	Total
DC1				1	2	4	3														10
DC2				2	4	3	1														10
DC3				2	3	1	4														10
DC10								1	2	1	2	1									7
DC10'								1	2	0	2	1									6
DC10''								2	1	1	1	1									6
DC15									3	1	2	1									7
DC15'									1	2	2	1									6
DC15''									1	1	2	1									5
DC20										1	1	2	1								5
DC20'											2	1	1								4
DC20''											1	1	1								3
CA1												1	2	2	1	1	1				8
CA2												2	1	1	1	2	1				8
CA3												1	1	1	3	1	2				9
CA10															1	1	2	1	1		6
CA10 '															1	2	1	1	1		6
CA10''																2	1	1	1		5
CA15																1	1	2	1		5
CA15'																	1	1	2		4
CA15''																	2	1	1		4
CA20																		1	2	1	4
CA20'																		1	1	1	3
CA20''																			1	1	2

Table S2. Allelopathic activity on shoot and root lengths of germinating seeds

		PLANT 1			PLANT 2			PLANT 3			PLANT 4			PLANT 5
concentration		S	R	L	S	R	L	S	R	L	S	R	L	S
DC1	10	8	2.5	2	7.5	3	2	6	4	2	4	6	2	7.5
DC2	10	6	2.5	2	3.8	1.7	2	6.1	4	2	3.8	5.7	2	7.3
DC3	10	4.5	3.5	2	5.5	5	2	5.3	6	2	6.3	7.2	2	6
DC10	7	6.1	2.2	2	5.1	4.2	2	3.5	4.2	2	4.2	5.5	2	5.6
DC10 '	6	4.2	2.1	2	4.6	4.1	2	3.1	4.5	2	5	5.2	2	5.5
DC10''	6	5	3.2	2	4.4	3.5	2	3.5	4.1	2	5.2	5.1	2	5.1
DC15	7	4	2	2	4.2	3.2	2	3.1	3.1	2	4.1	4.5	2	4.5
DC15'	6	3.7	1.2	2	4.1	2.8	2	2.7	2.7	2	3.7	4.1	2	4.7
DC15''	5	3.1	2	2	3.5	2.4	2	2.5	2.8	2	3.8	3.8	2	4.6
DC20	5	2.4	1.4	2	2.1	2.1	2	2	2.1	2	3.2	3.1	2	3.5
DC20'	4	2	1.5	2	2	2.4	2	1.5	1.8	2	2.5	2.6	2	3.5
DC20''	3	2.6	1.3	2	1.5	1.5	2	1.2	1.6	2	2.5	2.6	2	3.5
CA1	8	3.4	1.8	2	3.2	1.6	2	3.3	1.9	2	3.1	2	2	3.4
CA2	8	3.3	1.2	2	3.3	1.5	2	3.2	1.5	2	3.5	1.9	2	3.3
CA3	9	4	3.2	2	4	3.7	2	4	3	2	5	3	2	4.1
CA10	0	3.2	1.6	2	3.1	1.4	2	3.1	1.9	2	4	1.4	2	3.1
CA10'	0	3.1	1.2	2	3	1.7	2	3	1.5	2	3.5	1.5	2	3.1
CA10''	0	3.4	1.3	2	3.2	1.2	2	2.7	1.7	2	3.2	1.2	2	3.3
CA15	0	3.1	1.2	2	3	1.1	2	3.1	1.5	2	3.6	1	2	3
CA15'	0	3.1	1	2	2.6	1.1	2	2.4	1.4	2	3.1	1.1	2	3
CA15''	0	3	1.1	2	2.7	1	2	2.5	1.2	2	2.7	1	2	3
CA20	0	2.8	0.6	2	2.4	0.8	2	2.1	1.1	2	2.4	0.8	2	3
CA20'	0	2.4	0.4	2	2.2	0.6	2	2.1	0.8	2	2.4	0.8	2	3
CA20''	0	2.5	0.7	2	2.3	0.7	2	2.1	0.8	2	2.4	0.8	2	3
